# Single-cell analysis reveals an important role of CD137L^**+**^ macrophages in the host response to uropathogenic *Escherichia coli* infection in the bladder

**DOI:** 10.1371/journal.ppat.1013543

**Published:** 2025-10-03

**Authors:** Yaxiao Liu, Zizhuo Yang, Yinrui Xiang, Guangzhou Cheng, Lipeng Chen, Shuai Wang, Maolin Zang, Nan Zhou, Xiaoyi Zhang, Rui Chen, Benkang Shi, Yan Li

**Affiliations:** 1 Department of Urology, Qilu Hospital, Cheeloo College of Medicine, Shandong University, Jinan, Shandong, China; 2 Department of Urology, Shandong Provincial Hospital Affiliated to Shandong First Medical University, Jinan, Shandong, China; 3 Key Laboratory of Urinary Precision Diagnosis and Treatment in Universities of Shandong, Jinan, Shandong, China; 4 Department of Urology, Tengzhou Central People’s Hospital, Tengzhou, Shandong, China; 5 Shenzhen Research Institute of Shandong University, Shenzhen, China; Universite Paris Descartes Faculte de Medecine, FRANCE

## Abstract

Uropathogenic *Escherichia coli* (UPEC) typically trigger rapid and robust innate immune responses in the bladder. In order to identify the key facets of the host response that influence pathogen clearance and tissue damage, single-cell RNA sequencing was used to investigate the transcriptomic changes of immune cells in mouse bladder after UPEC infection. Single-cell analysis revealed significant elevated CD137L expression in macrophages and dendritic cells in bladder after UPEC infection. CD137L defines a macrophage population in bladder that is important for the host response to UPEC infection. Deletion of CD137L in macrophages resulted in severe bacterial burden and bladder inflammation during the acute stage of UPEC infection. Further study demonstrated that the crucial role of CD137L^+^ macrophages in protecting against UPEC infection might be mediated by Tregs, which express high levels of CD137 (the receptor for CD137L). Deletion of CD137L^+^ macrophages decreased Treg cells and led to a reduction in inhibitory factors such as CTLA-4 and PD-1 on Tregs. Deletion of Tregs using Foxp3DTR mice also aggravated inflammatory reactions, bacterial load, and urothelial destruction during the acute phase of UPEC infection. Similarly, the deletion of CD137 in Tregs resulted in a decrease in these inhibitory factors on Tregs, causing more severe bladder inflammation during UPEC infection. These results illuminate the immune landscape of the bladder infected by UPEC and highlight the crucial role of CD137L^+^ macrophages during UPEC infection in bladder. CD137L^+^ macrophages might prevent excessive inflammatory response during the host response to UPEC infection by regulating Tregs.

## Author summary

Precise regulation of host immune and inflammatory processes is essential for restricting UPEC pathogenesis in the bladder. Host resistance to infection relies on tightly regulated immune responses. In this study, we utilized scRNA-seq, along with pathway enrichment analysis and intercellular interaction analysis, to profile an unbiased immune landscape of the bladder after UPEC infection. We identified a population of CD137L^+^macrophages that play an important role in the host response to uropathogenic Escherichia coli infection in bladder. Deletion of CD137L on macrophages led to the aggravation of CFU burden and bladder inflammation during UPEC infection. The crucial role of CD137L^+^ macrophages in protecting against UPEC infection might be mediated by Tregs, which prevented excessive inflammatory response during the host response to UPEC infection.

## Introduction

Urinary tract infections (UTIs) are a major global health burden, affecting more than 150 million people annually [1]. The primary causative agents are bacteria, with uropathogenic *Escherichia coli* (UPEC) responsible for 70–80% of cases. Approximately half of all women experience a UTI in their lifetime, and many develop recurrent or chronic infections that significantly impair quality of life [2,3]. UTIs are classified by anatomical site: lower UTIs (cystitis, involving the bladder) and upper UTIs (pyelonephritis, involving the kidney). Unlike pyelonephritis or many other infectious diseases, bladder infection only evokes a minimal pathogen-specific antibody response and rarely cause systemic symptoms [4–6]. Additionally, cystitis recurs far more frequently than upper UTIs. These distinct features highlight the unique nature of the local immune response to UPEC within the bladder. Gaining a deeper understanding of this specific bladder immunity is therefore crucial for developing more effective strategies to manage and prevent cystitis.

In bladder, a large number of immune cell types, including macrophages, the dendritic cells, T cells, eosinophils, natural killer cells and mast cells, reside in the naive bladder. Despite the high incidence of cystitis, there is a relative scarcity of data profiling bladder-resident immune cells. Recent studies have provided some insights into the functions of immune cells, especially for macrophages, mast cells and CD4^+^ T helper cells in the host response to bladder infection [4,7–9]. Macrophages are a critical component of anti-UTI defense. During infection, pathogen-associated and damage-associated molecular patterns (PAMPs and DAMPs) released into the urinary space and surrounding tissues can influence both resident macrophages and recruited Ly6C^+^ monocytes, driving them towards pro-inflammatory (“M1-like”) or alternatively activated (“M2-like”, M2a, M2b, M2c) phenotypes [10,11]. Notably, recent advances highlight the substantial plasticity and fluidity of macrophage responses to PAMPs/DAMPs, indicating that the traditional M1/M2 paradigm is overly rigid [12]. Therefore, investigating more nuanced macrophage subpopulations is crucial for UTI prevention and treatment.

While bacterial infection triggers rapid bladder inflammation but often fails to activate robust adaptive immunity or generate sufficient pathogen-specific antibodies [13]. This suggests the presence of unidentified immunosuppressive mechanisms that likely function to curb excessive inflammation and facilitate rapid restoration of the urothelial barrier. Importantly, uncontrolled immune activation can cause severe inflammation and epithelial damage, increasing UTI susceptibility. Consequently, precise regulation of host immune and inflammatory processes is key to limiting UPEC pathogenesis [14]. Regulatory T cells (Tregs), pivotal immunosuppressive players in the immune regulatory network, are increasingly recognized for their critical role in modulating pathogen-induced inflammation [15]. For example, commensal bacteria effectively induce Treg differentiation in the colon [16]; probiotics influence the Treg/Th17 balance to protect lung epithelial homeostasis during injury [17]; and CD301b^+^ dendritic cells specifically capture and present bacterial antigens to induce Tregs regulating the skin barrier [18]. However, the immunomodulatory role of Tregs in UTIs remains unexplored.

To further gain a comprehensive understanding of the immune microenvironment regulation during bladder infection, we established a murine model of UPEC infection, and CD45^+^ cells in bladder were harvested for single-cell RNA sequencing (scRNA-seq) 24h after UPEC infection. scRNA-seq analysis revealed an unbiased picture of immune cell diversity and functional states in the bladder during UPEC infection. Significantly elevated expression of *Tnfsf9*, the gene encoding CD137L, was identified specifically in antigen-presenting cells (APCs) 24h after UPEC infection in bladder. Further study found that CD137L^+^macrophages, as opposed to CD137L^+^dendritic cells, play a important role in protecting safeguarding the bladder against UPEC infection. Additionally, our findings demonstrate that CD137^+^Tregs, which was regulated by CD137L^+^ macrophages, play pivotal roles in prevent excessive inflammation during the host response to UPEC infection.

## Materials and methods

### Ethics statement

Ethical approval was obtained from the Laboratory Animal Ethics and Welfare Committee of Cheeloo College of Medicine, Shandong University (Approval No. 22027).

#### Murine UTIs model:.

Female C57BL/6 mice (8-week-old) were purchased from Beijing Vital River Laboratory Animal Technology Co. Ltd.. *Lyz2*^Cre^ mice, *Foxp3*^Cre^ mice, *Tnfsf9*^F/F^ mice and *Tnfrsf*9^F/F^mice were purchased from GemPharmatech Co. Ltd.. *Foxp3-*DTR mice were purchased from the Jackson Laboratory. All mice were maintained in the specific pathogen-free animal facility of our institution. Uropathogenic *Escherichia coli* (UPEC) strain CFT073 was purchased from American type culture collection (700928). For depletion of Tregs, 1 μg diphtheria toxin (Listlabs) was injected intraperitoneally on 3 consecutive days before UTIs. Mice in the UPEC group were anesthetized and subjected to cystitis induction via transurethral instillation of 30 μL UPEC suspension (1–2 × 10^7^ colony-forming units (CFU) in PBS) using a mouse-specific catheter (inner diameter: 0.28 mm). Mice in the Control group were received an equivalent volume (30 μL) of PBS via bladder instillation. [19].

#### Preparation of single-cell suspensions:.

Mice were euthanized by cervical dislocation and bladders were removed. Bladders were washed with cold phosphate buffered saline (PBS), dissected, minced, and incubated in 20 mg/ml dispase II, 1 mg/ml DNaseI and 0.5 mg/ml Liberase TL solution at 37°C for 2 hours. The digested tissue was vigorously shaken to homogenize it and filtered through a 100-μm cell strainer. Mononuclear cells were then isolated by density gradient centrifugation using Percoll (80% over 40%) and harvested from the interface after centrifugation at 2500 rpm for 20 min at room temperature. To block non-specific Fc receptor binding, cells were treated with CD16/32 antibody prior to staining with CD45.2 antibody. Cell viability was assessed using the LIVE/DEAD Violet Viability Kit Live CD45^+^ single-cell suspensions were finally isolated/purified by FACS.

#### Single-cell RNA-Sequencing data processing, quality control, and batch effect adjustment:.

Single-cell suspensions were loaded onto microfluidic devices, and scRNA-seq libraries were constructed according to the Singleron GEXSCOPE protocol in the GEXSCOPE Single-Cell RNA Library Kit. Raw reads were processed with fastQC and fastp to remove low-quality reads. Unique molecular identifiers (UMI) and cell barcodes were extracted after filtering out reads without poly-A tails. Poly-A tails and adapter sequences were then trimmed by cutadapt, and the reads that met quality control standards were matched to the reference genome mm10 using STAR. Doublets of scRNA-seq were excluded by first using DoubletFinder. Gene counts and UMI counts were grouped by feature Counts software to generate expression matrix files for downstream analyses. We apply a criterion to filter out cells with UMI/gene numbers out of the limit of mean value + / − 2-fold of standard deviations assuming a Guassian distribution of each cells’ UMI/gene numbers. And cells with more than 7.5% mitochondrial and 20% ribosomal content were removed.

#### Dimensional reduction and clustering cell type Identification:.

Then Seurat v2.3 was used for dimension-reduction and clustering. NormalizeData and ScaleData were used to normalize and scale all gene expression values. The top 2000 variable genes were selected for principal component analysis (PCA) by FindVariableFeatures. Then we used the harmony R package to perform batch effects adjustment. The resolution was then set to 0.6 to isolate subcluster cell types. A UMAP algorithm was performed to visualize the cells in two-dimensional space. The cell type identification of each cluster was manually annotated according to the expression of canonical markers.

#### Pathway enrichment analysis:.

Gene Ontology (GO) analyses were performed using the “clusterProfiler” R package. Pathways with a P value of less than 0.05 were considered significantly enriched. Gene Set Variation Analysis (GSVA) was conducted to assess the differences in biological pathways between subgroups using the GSVA R package, so as to evaluate whether the mechanism pathway is enriched among different clusters. Gene set enrichment analysis (GSEA) analyses were used to evaluate the enrichment of a prior defined sets of genes associated with particular biological processes by fgsea R package.

#### Cell–cell interaction analysis (CellChat):.

CellChat analyses were used to quantitatively infer and analyses intercellular communication networks from single cell RNA-seq data.

#### HE staining and Immunofluorescence:.

Bladder tissues from mice were fixed in 10% formalin and embedded in paraffin. Paraffin sections underwent hematoxylin and eosin (H&E) staining. Histological scoring was performed based on parameters including edema, bleeding, inflammatory cell infiltration, and epithelial changes, with scores ranging from 0 to 5, as previously described [20]. The bladder sections were incubated overnight at 4°C with an anti-Uroplakin3a (UPK3a) primary antibody. After washing with PBS, the slides were incubated at room temperature for 1 hour with an Alexa Fluor 488-conjugated secondary antibody. Following PBS washes, slides were counterstained with DAPI for 10 minutes at room temperature. Finally, slides were mounted with an anti-fade mounting medium and visualized using a fluorescence microscope.

#### Flow cytometry:.

Preparation of immune cell suspensions was described above. Surface antigens were stained and fixation and permeabilization. Then stained intracellular proteins. Antibodies used for staining: CD45.2-AF700: Invitrogen Cat#56-0454-82; CD45.2-PE: Biolegend 109808; CD11b-PerCP/Cyanine5.5: Biolegend Cat#101228; Ly-6G-FITC: Biolegend 164508; F4/80-PE: Biolegend Cat#123110; CD137L-APC: Invitrogen Cat#MA5–46776; MHCII-PerCP/Cyanine5.5: Biolegend 107626; Ly6C-PB: Biolegend Cat#128013; CD3-PerCP/Cyanine5.5: Biolegend 100218; CD3-APC: Biolegend 100236; CD4-APC/Cyanine7: Biolegend Cat#100414; TCR-β-PerCP/Cyanine5.5: Biolegend 109228; TCR-β-FITC: Biolegend 159706; Foxp3-PB: Invitrogen Cat#48-5773-82; CTLA-4-PE: Biolegend 106306; PD-1-PECy7: Biolegend 135216; CD137-PE/Cyanine7: Invitrogen Cat#25-1371-82. Flow cytometry was conducted using Gallios flow cytometry (Beckman). Data were analyzed FlowJo software.

#### Bacterial load assessment:.

The bladders were collected and homogenized in 0.1% Triton X-100. Serial dilution of bacteria was plated on LB agar and CFU were counted. Each experiment was repeated three times with three technical replicates of each condition in each assay.

#### RT-PCR analysis:.

Total RNA was extracted by using TRAzol according to the manufacturer’s instructions. Extracted RNA was synthesized into cDNA using PrimeScript RT Master Mix. Synthesized cDNA was used for RT-PCR with SYBR Green Pro Taq Mix following the manufacturer’s instructions. The result of mRNA relative expression levels was analyzed by the 2^−ΔΔCt^ method. GAPDH was used as the referenced gene. The following primers were used:

mus-*Il1b*-F:5’-GTGTCTTTCCCGTGGACCTT-3’,
mus-*Il1b*-R: 5’-AATGGGAACGTCACACACCA-3’;
mus-*Il6*-F: 5’-CTTCTTGGGACTGATGCTGGT-3’,
mus-*Il6*-R: 5’-CTCTGTGAAGTCTCCTCTCCG-3’;
mus-*Tnf*-F: 5’-AGCCGATGGGTTGTACCTTG-3’,
mus-*Tnf*-R: 5’-ATAGCAAATCGGCTGACGGT-3’.

#### Statistics:.

Data were analyzed by GraphPad Prism software. A two-tailed unpaired Student’s t-test was used for comparison between the two groups. **P* *< 0.05 was considered to be significant. *: **p* *< 0.05, **: **p* *< 0.01, ***: **p* *< 0.001, ****: **p* *< 0.0001.

### Data and materials availability

All data needed to evaluate the conclusions in the paper are present in the paper and/or the Supplementary Materials. All sequencing data is available at GEO GSE252321.

## Results

### scRNA-seq analysis identified a group of CD137L^+^macrophages in bladder after UPEC infection

**Fig 1:**
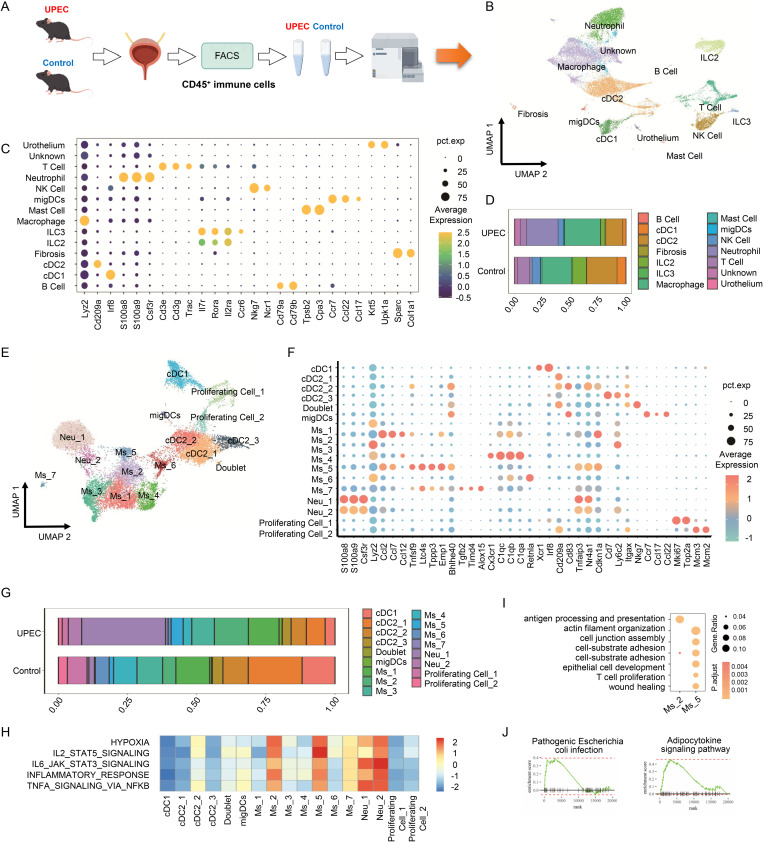
CD137L^+^macrophages increase in the bladder during UPEC infection. **(A)** Graphical illustration of the experimental setup. Bladders were harvested from both UPEC and control groups (in duplicate), then enzymatically digested to generate single-cell suspensions. Live CD45^+^ cells were FACS-sorted and loaded cells for scRNA-seq. This figure was created using Figdraw and is licensed with authorization from Figdraw (www.figdraw.com). **(B)** Nonlinear dimensionality reduction Uniform Manifold Approximation (UMAP) visualization of 24415 bladder CD45^+^ immune cells identified 13 different cell types after unsupervised clustering in healthy and UPEC infection group, respectively. Each point depicts a single cell, colored according to cell type designation. **(C)** Dot plots of gene expression level identified within CD45^+^ immune cell populations. **(D)** Summary of proportion of assigned cell types in healthy and UPEC infection group, respectively. **(E)** UMAP visualization of 16 distinct bladder monocyte clusters, myeloid populations colored in accordance of group. **(F)** Dot plots of gene expression level identified within myeloid populations. **(G)** Bar graph of relative abundance of each cluster of myeloid types healthy and UPEC infection conditions. **(H)** Differences in selected hallmark pathway activities scored with GSVA software. **(I)** Dot plots displaying the representative differentially enriched GOBP terms between Ms_2 (*Ccl2*^hi^Ms) and Ms_5 (*Tnfsf9*^+^Ms). **(J)** GSEA reveals bacterial infection associated signal pathways enriched in Ms_5 (*Tnfsf9*^+^Ms) compared with Ms_2 (*Ccl2*^hi^Ms).

We performed scRNA-seq on CD45^+^ cells harvested from female mouse bladders 24h after UPEC infection (Figs 1A and S1A). The PBS control group dataset was obtained from our previously published study [21], generated under identical experimental conditions. The UPEC-infected and PBS control datasets were jointly processed using a common preprocessing and visualization pipeline (Uniform Manifold Approximation and Projection, UMAP) to map the diverse immune cell landscape in both groups (Fig 1B). After filtering out low-quality cells (S2 Fig), a total of 24,415 high-quality cells were obtained for further analysis, with 19,612 features detected. Unsupervised clustering identified 22 distinct cell clusters (Fig 1C). In addition to immune cells, fibroblast and urothelial cells were also identified, likely as a result of technical constraints. Changes in immune cell composition were observed between the Control and UPEC-infected groups (Fig 1D). Consistent with prior studies, the immune response to UPEC infection in the bladder is characterized by robust cytokine expression, driving rapid infiltration of substantial a number of neutrophils (increasing from 2.18% in Controls to 28.21% in UPEC) and macrophages (increasing from 26.25% to 32.25%) [22,23]. Next, we reclustered myeloid cells (neutrophils, DCs and macrophages) (Fig 1E). This analysis identified 2 transcriptionally distinct neutrophil subpopulations (Neu_1 and Neu_2), 3 distinct cDC2 subpopulations (cDC2_1–3), 1 group of cDC1, and 7 distinct macrophage subpopulations (Ms_1–7), along with 2 groups of proliferating cells (Fig 1F).

Proportions of Ms_2, Ms_5 and Ms_7 were significantly elevated in the UPEC group compared to the Control group (Fig 1G). Ms_2, designated *Ccl2*^hi^Ms, exhibited high expression of *Ccl2*, *Ccl7* and *Ccl12*, which were associated with response to biotic stimulus and regulation of monocyte/macrophage migration [24]. Ms_5, designated *Tnfsf9*^+^Ms, characterized by elevated *Tnfsf9* (encoding CD137L), *Ccl2*, *Ltc4s*, *Tppp3*, *Emp1* and *Bhlhe40*, which were linked to immune cell recruitment during microbial infections and tissue damage [25]. Ms_7 (*Saa3*^+^Ms) showed high expression of *Saa3*, *Tgfb2*, *Timd4* and *Alox15*, genes implicated in halting leukocyte trafficking [26,27] and the recognition/phagocytosis of inflammatory cells [28,29]. GSVA further elucidated functional heterogeneity among these myeloid cell clusters (Fig 1H). Based on the expression of inflammatory signaling pathways such as ‘Inflammation response’, ‘TNFα signaling via NF‐κB’ and ‘IL6 JAK STAT3 signaling’, we classified *Ccl2*^hi^Ms and *Tnfsf9*^+^Ms as inflammation-Ms cells. To delineate their distinct functions, GO analysis was performed on *Ccl2*^hi^Ms and *Tnfsf9*^+^Ms. This revealed shared enrichment for ‘myeloid cell differentiation’ and ‘leukocyte migration’. However, distinct functional profiles emerged: *Tnfsf*9^+^Ms were uniquely enriched for processes including ‘wound healing’, ‘T cell proliferation’, ‘epithelial cell development’, and ‘actin filament organization’, whereas *Ccl2*^hi^Ms showed specific enrichment for ‘antigen processing and presentation’ (Fig 1I). Furthermore, GSEA indicated that *Tnfsf9*^+^Ms exhibited significant enrichment for multiple signaling pathways associated with bacterial infection compared to *Ccl2*^hi^Ms (Fig 1J). Collectively, these findings indicate that CD137L^+^ macrophages play a crucial role in the immune response against UPEC infection.

### Deletion of CD137L on macrophages exacerbated UPEC burden and bladder inflammation during the acute UPEC infection

**Fig 2:**
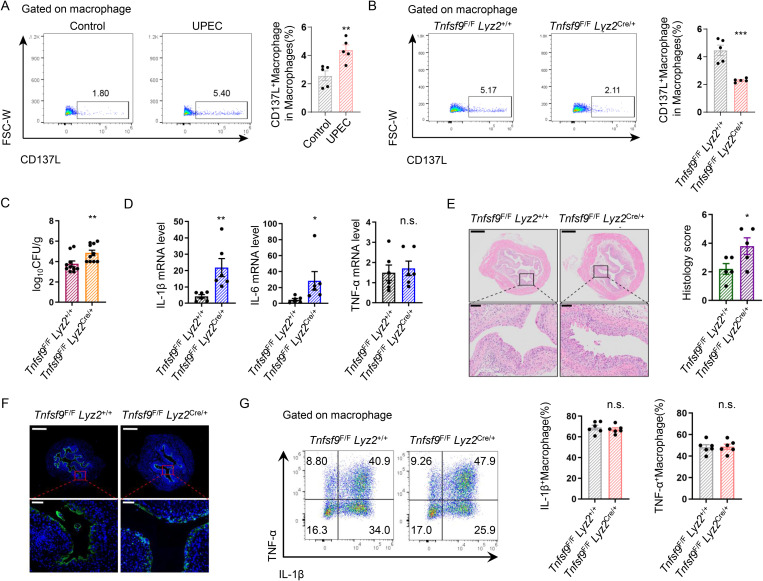
Deletion of CD137L on macrophages aggravated UPEC burden and bladder inflammation during UPEC infection. **(A)**CD137L^+^Ms in bladders from 8-week-old mice in UPEC and Control groups were analyzed by flow cytometry. Dot plots depict the gating strategy for CD137L^+^Ms. Graph shows the proportion of bladder CD137L^+^Ms (*n* = 5). **(B)** CD137L^+^Ms in bladders from 8-week-old infected *Tnfsf9*^F/F^ *Lyz2*^+/+^ and *Tnfsf9*^F/F^ *Lyz2*^Cre/+^ mice were analyzed by flow cytometry. Dot plots depict the gating strategy for CD137L ^+^Ms. Graph shows the proportion of bladder CD137L^+^Ms (n = 5). **(C)** Bacterial load was assessed 24 hours after infection (*n* = 10). **(D)** The mRNA expression of *Il1b*, *Il6* and *Tnf* in the bladder tissues were measured by RT-PCR (*n* = 6). **(E)** H&E Staining of bladders from 8-week-old female *Tnfsf9*^F/F^ *Lyz2*^+/+^ and *Tnfsf9*^F/F^ *Lyz2*^Cre/+^ mice. Histology scores were assessed after infection (*n* = 5). **(F)** Representative images of uroplakin3a in superficial bladder urothelium of *Tnfsf9*^F/F^ *Lyz2*^+/+^ and *Tnfsf9*^F/F^ *Lyz2*^Cre/+^ UTIs mice models (*n* = 4). **(G)** The expression of IL-1β and TNF-α on macrophages between infected *Tnfsf9*^F/F^ *Lyz2*^+/+^ and *Tnfsf9*^F/F^ *Lyz2*^Cre/+^ mice were analyzed by flow cytometry. The graph depicts the expression of IL-1β and TNF-α on macrophages (n = 6).

Building on previous reports implicating CD137L in inflammation progression [30,31]. we further examined the role of CD137L^+^ macrophages during UPEC infection. Macrophage-specific deletion of CD137L was achieved using *Tnfsf9*^F/F^ *Lyz2*^Cre/+^ mice (Figs 2A, 2B and S1B). At 24 hours post-infection, *Tnfsf9*^F/F^ *Lyz2*^Cre/+^ mice exhibited increased CFU burden (Fig 2C) and elevated IL-1β and IL-6 expression relative to *Tnfsf9*^F/F^ *Lyz2*^+/+^ mice, with no significant difference in *Tnf* (Fig 2D). Histopathological analysis showed more severe bladder inflammation in *Tnfsf9*^F/F^ *Lyz2*^Cre/+^ mice versus *Tnfsf9*^F/F^ *Lyz2*^+/+^ mice (Fig 2E). Immunofluorescence staining of uroplakins revealed incomplete umbrella cell structure following UPEC infection, which was significantly exacerbated in *Tnfsf9*^F/F^ *Lyz2*^Cre/+^ mice (Fig 2F). Macrophages are known to play a pivotal role in bacterial cystitis, primarily through IL-1β, IL-6 and TNF-α secretion [32]. Additionally, some studies suggest that CD137L signaling can synergistically enhance inflammatory mediator production in macrophages alongside other stimuli [33]. However, we detected no differences in macrophage IL-1β and TNF-α expression (no IL-6 expression detected on macrophages) on macrophages between *Tnfsf9*^F/F^ *Lyz2*^cre/+^ and *Tnfsf9*^F/F^ *Lyz2*^+/+^ mice (Fig 2G). Consequently, we infer that the exacerbation of inflammation caused by macrophage-specific CD137L deletion is not attributable to altered IL-1β/TNF-α production in macrophages.

**Fig 3:**
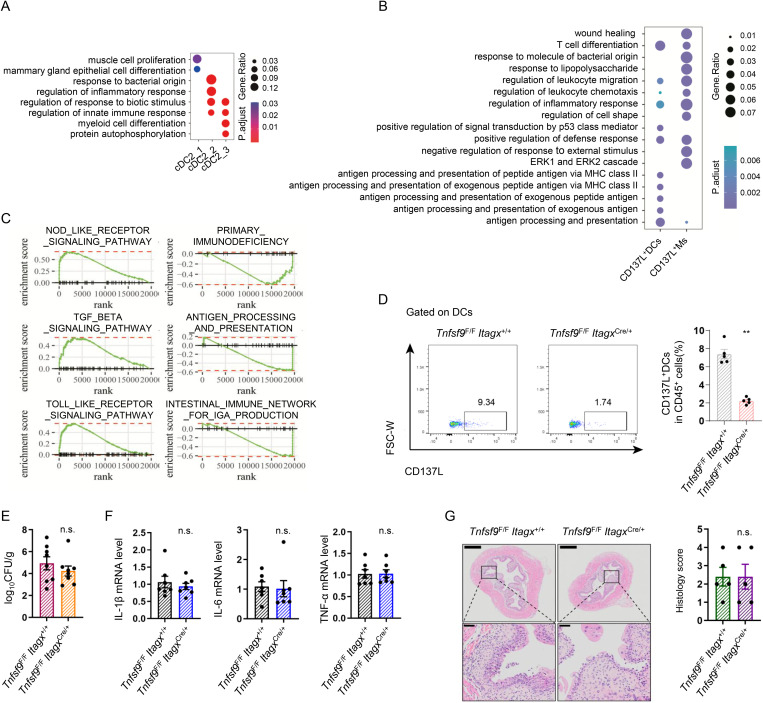
Deletion of CD137L on DCs does not impact UPEC burden and bladder inflammation during UPEC infection. **(A)** Dot plots displaying the representative differentially enriched GOBP terms among cDC2 populations. **(B)** Dot plots displaying the representative differentially enriched GOBP terms between CD137L^+^DCs and CD137L^+^Ms. **(C)** GSEA reveals infection and inflammation-related pathways enriched in CD137L^+^Ms compared with CD137L^+^DCs. **(D)** CD137L^+^DCs in bladders from 8-week-old infected *Tnfsf9*^F/F^ *Itagx*^+/+^ and *Tnfsf9*^F/F^ *Itagx*^Cre/+^ mice were analyzed by flow cytometry. Dot plots depict the gating strategy for CD137L^+^DCs. Graph shows the proportion of bladder CD137L^+^DCs (n = 5). **(E)** Bacterial load was assessed 24 hours after infection (*n* = 8). **(F)** The mRNA expression of *Il1b*, *Il6* and *Tnf* in the bladder tissues were measured by RT-PCR (*n* = 6). **(G)** H&E Staining of bladders from 8-week-old female *Tnfsf9*^F/F^ *Itagx*^+/+^ and *Tnfsf9*^F/F^ *Itagx*^Cre/+^ mice. Histology scores were assessed after infection (*n* = 5).

Notably, in addition to macrophages, cDC2_2 also exhibited higher *Tnfsf9* expression than other cDC2 clusters. Differential expression analysis revealed that the cells of the cDC2_2 express higher levels of maturation/activation markers required for cDC2 stimulation (*Cd83*), inflammation-regulating molecules (*Tnfaip3* and *Nr4a1*) and adhesion molecules (*Cdkn1a*) compared to other cDC2 [34–36]. GO analysis of cDC2_2 cluster markers identified enrichment for biological processes related to ‘response to bacterial origin’ and ‘regulation of inflammatory response’ (Fig 3A). We further investigated CD137L^+^DCs function during UPEC infection. scRNA-seq analysis indicated that CD137L^+^macrophages were primarily associated with “wound healing”, “response to molecule of bacterial origin” and “negative regulation of response to external stimulus”, whereas CD137L^+^DCs were predominantly linked to “antigen processing and presentation” (Fig 3B). GSEA of CD137L^+^ macrophages marker genes demonstrated significant enrichment for multiple infection and inflammation-related pathways compared with CD137L^+^DCs, most notably the ‘NOD-like receptor signaling pathway’ and ‘Toll like receptor signaling pathway’ (Fig 3C), suggesting a more critical role for CD137L^+^ macrophages in acute bladder infection. While Lyz2-Cre mice are frequently used to target macrophages, the Lyz2 promoter drives Cre recombinase expression broadly in myeloid cells, including DCs. Therefore, the observed phenotype in *Tnfsf9*^F/F^ *Lyz2*^Cre/+^ mice could reflect the combined loss of CD137L in both macrophages and DCs. To specifically delineate the function of CD137L^+^ macrophages, we employed *Tnfsf9*^F/F^ *Itgax*^Cre/+^ mice to delete CD137L specifically in DCs (Fig 3D). *Tnfsf9*^F/F^ *Itgax*^Cre/+^ mice showed no differences from *Tnfsf9*^F/F^ *Itgax*^+/+^ mice in CFU burden, pro-inflammatory cytokine expression, or histopathological scores (Fig 3E-G). These data establish CD137L^+^ macrophage, but not CD137L^+^ DCs, as essential mediators of the host defense against UPEC bladder infection.

### Tregs in bladder, which express high levels of CD137, play a protective role during UPEC infection

**Fig 4:**
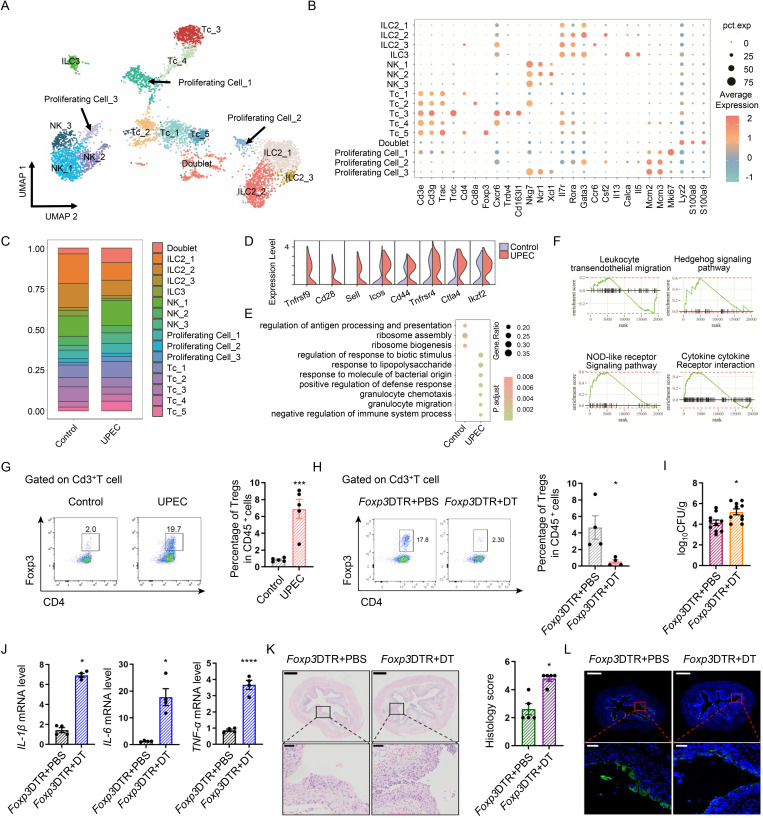
Tregs, which express high levels of CD137, play a protective role during UTIs. **(A)** UMAP visualization of 16 distinct bladder lymphoid cells in UPEC and Control groups, colored according to cluster designation. **(B)** Dot plots of gene expression level identified within lymphoid cells. **(C)** Summary of proportion of assigned lymphoid cells in UPEC and Control groups, respectively. **(D)** Representative genes related to typical functions of Tregs in UPEC and Control groups. **(E)** Dot plots displaying the representative differentially enriched GOBP terms between Tregs taken before and after the infection. **(F)** GSEA reveals infection-related pathways enriched in Tregs after UPEC infection. Each dot represents one mouse; lines are medians. **(G)** Tregs in bladders from 8-week-old mice in UPEC and Control groups were analyzed by flow cytometry. Graph shows the proportion of bladder Tregs (*n* = 5). **(H)** Tregs in bladder of *Foxp3*DTR with DT injection or not were analyzed by flow cytometry. Graph shows the proportion of bladder Tregs. Dot plots depict the gating strategy for Tregs (*n* = 4). **(I)** Bacterial load was assessed 24 hours after infection (*n* = 10). **(J)** The mRNA expression of *Il1b*, *Il6* and *Tnf* in the bladder tissues were measured by RT-PCR (*n* = 4). **(K)** H&E Staining of bladders from *Foxp3-*DTR and normal mice after UPEC infection. Histology scores were assessed after infection (*n* = 5). **(L)** Representative images of uroplakin3a in superficial bladder urothelium of *Foxp3*DTR and normal UTIs mice models (**n* *= 4).

Given reports that lymphocytes express high levels of CD137 (the receptor for CD137L) [37–39], we performed lymphocyte re-clustering. This identified 3 main lymphoid clusters (T Cells, NK Cells, ILCs) and 4 proliferating cell groups (Fig 4A). The T cell compartment comprised five subsets: Tc_1 (CD4^+^ T Cells), Tc_2 (CD8^+^T Cells), Tc_3 (*Cxcr6*^+^γδT resident memory T cells expressing *Trpv4* and *Cd163l*), Tc_4 (*Cxcr6*^+^αβT esident memory T cells expressing *Lmo4* and *Rexo2*), and Tc_5 (Tregs) (Fig 4B and 4C). In Tregs, UPEC infection significantly elevated expression of *Tnfrsf9* (CD137), *Cd28*, *Icos*, and *Ctla4* versus controls (Fig 4D). GO enrichment analysis revealed UPEC-exposed Tregs were enriched for ‘processes of innate immune regulation’, ‘granulocyte migration’ and ‘response to molecule of bacterial origin’ (Fig 4E). GSEA revealed several infection-related pathways of Tregs after UPEC infection were upregulated, which could be associated with an activated state in response to exposure to UPEC (Fig 4F). Flow cytometry confirmed that UPEC-infected bladders exhibit an increase in Tregs compared with naive bladders (Figs 4G and S1D). To further validate our hypothesis, we used *Foxp3*DTR mice to selectively deplete Tregs through diphtheria toxin (DT) injection (Fig 4H). Treg ablation exacerbated infection severity, including increased CFU burden (Fig 4I), elevated pro-inflammatory cytokines (Fig 4J), higher histopathological score (Fig 4K) and worsened epithelial exfoliation (Fig 4L). These findings establish Tregs as critical modulators of UPEC-induced bladder pathology.

### CD137L^+^macrophages play an important role in maintaining the inhibitory function of Tregs

**Fig 5:**
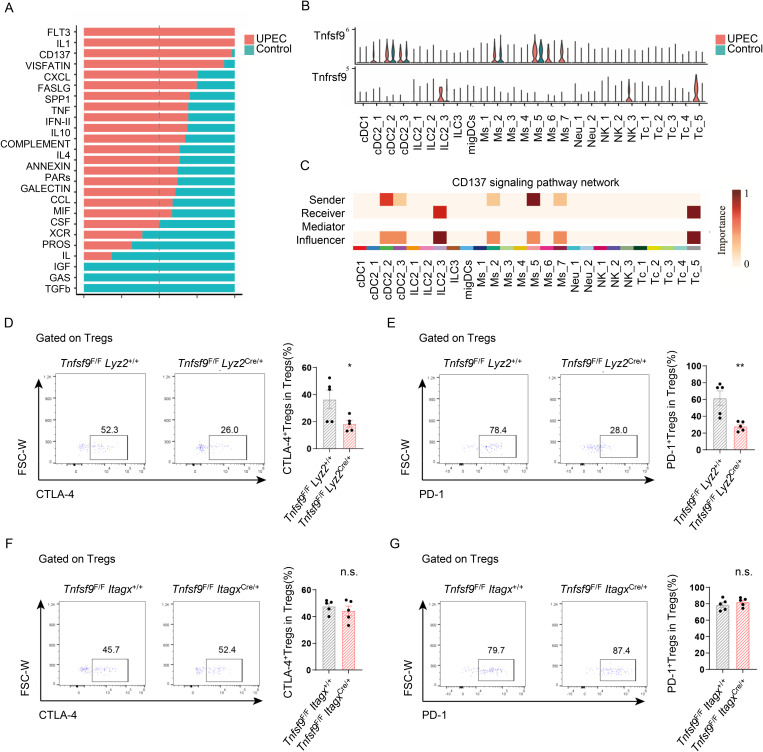
CD137L^+^macrophages play an important role in maintaining the inhibitory function of Tregs. **(A)** Selected significant signaling pathways were ranked based on their differences in overall information flow within the inferred networks between UPEC and Control groups. The top signaling pathways colored red are more enriched in UPEC group, the middle one colored black is equally enriched in UPEC and Control groups, and the bottom ones colored green are more enriched in Control group. **(B)** Violin plot showing the expression distribution of signaling genes involved in the inferred CD137 signaling network in UPEC and Control groups. **(C)** Heatmap shows the relative importance of each cell group based on the computed four network centrality measures of CD137 signaling network. **(D-E)** The expression of CTLA-4 and PD-1 on Tregs between infected *Tnfsf9*^F/F^ *Lyz2*^+/+^ and *Tnfsf9*^F/F^ *Lyz2*^Cre/+^ mice were analyzed by flow cytometry. The graph depicts the expression of CTLA-4 and PD-1 on Tregs (n = 5). **(F-G)** The expression of CTLA-4 and PD-1 on Tregs between infected *Tnfsf9*^F/F^ *Itagx*^+/+^ and *Tnfsf9*^F/F^ *Itagx*^Cre/+^ mice were analyzed by flow cytometry. The graph depicts the expression of CTLA-4 and PD-1 on Tregs (n = 5).

Cells interact and communicate with each other through ligand-receptor pairs that coordinate many biological processes in both Control and UPEC group, as revealed by CellChat analysis. Comparing signaling pathway activity between these groups identified 14 pathways significantly upregulated during infection. These upregulated pathways, including CD137, IL10, IL1, and VISFATIN, are primarily immune-related, indicating that enhanced proinflammatory signaling critically contributes to disease progression (Fig 5A). Notably, UPEC infection markedly altered communication between CD137L⁺ macrophages and Tregs. Focusing specifically on CD137 (*Tnfrsf9*) signaling, CellChat pinpointed CD137L-CD137 as a key interaction pathway linking these cell types in infected mice (Fig 5B). Dendritic cells and macrophages serve as the primary sources of the signal, while ILC2s and Tregs function as the primary recipients (Fig 5C). It is known that Tregs primarily exert their inhibitory effects by secreting immunosuppressive factors such as IL10 and TGFβ or by expressing CTLA-4 and PD-1. Through flow cytometry, we observed that the deletion of CD137L^+^macrophages reduces the inhibitory factors such as CTLA-4 and PD-1 on Tregs (Fig 5D and 5E) (no IL-10 and TGF-β expression detected on Tregs). In contrast, depletion of CD137L ⁺ DC did not affect CTLA-4 or PD-1 expression on Tregs (Fig 5F and 5G). Collectively, these findings demonstrate a crucial role for bladder CD137L⁺ macrophages in maintaining Treg immunosuppressive function during UPEC infection.

### Deletion of CD137 on Tregs aggravated UPEC burden and bladder inflammation during UPEC infection in the bladder

**Fig 6:**
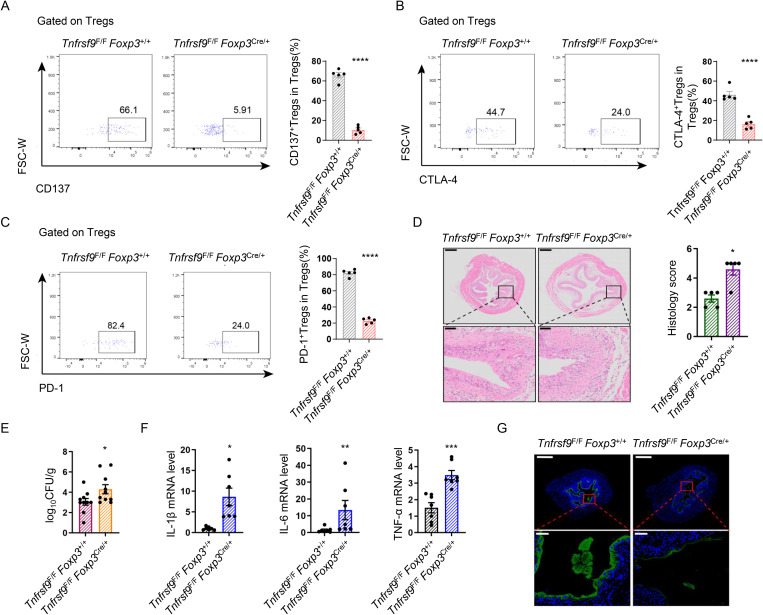
Deletion of CD137 on Tregs aggravated UPEC burden and bladder inflammation during UPEC infection in the bladder. **(A)** CD137^+^Tregs in bladders from 8-week-old infected *Tnfrsf9*^F/F^ *Foxp3*^+/+^ and *Tnfrsf9*^F/F^ *Foxp3*^Cre/+^ mice were analyzed by flow cytometry. Dot plots depict the gating strategy for CD137L ^+^DCs. Graph shows the proportion of bladder CD137L^+^DCs (n = 5). **(B-C)** The expression of CTLA-4 and PD-1 on Tregs between infected *Tnfrsf9*^F/F^ *Foxp3*^+/+^ and *Tnfrsf9*^F/F^ *Foxp3*^Cre/+^ mice were analyzed by flow cytometry. The graph depicts the expression of CTLA-4 and CCC on Tregs (n = 5). **(D)** H&E Staining of bladders from 8-week-old female *Tnfrsf9*^F/F^ *Foxp3*^+/+^ and *Tnfrsf9*^F/F^ *Foxp3*^Cre/+^ mice. Histology scores were assessed after infection (*n* = 5). **(E)** Bacterial load was assessed 24 hours after infection (*n* = 10). **(F)** The mRNA expression of *Il1b*, *Il6* and *Tnf* in the bladder tissues were measured by RT-PCR (*n* = 6). **(G)** Representative images of uroplakin3a in superficial bladder urothelium of *Tnfrsf9*^F/F^ *Foxp3*^+/+^ and *Tnfrsf9*^F/F^ *Foxp3*^Cre/+^ mice UTIs mice models (*n* = 4).

To further validate the role of CD137 signaling in Tregs, we used *Tnfrsf9*^F/F^*Foxp3*^cre/+^ mice to selectively deplete CD137 on Tregs (Fig 6A). Knockdown of CD137 on Treg reduced the expression of inhibitory factors such as CTLA-4 and PD-1 (Fig 6B and 6C). Compared to *Tnfrsf9*^F/F^*Foxp3*^+/+^ controls, *Tnfrsf9*^F/F^*Foxp3*^cre/+^mice developed more severe tissue edema and inflammation mice at the early stage of UPEC infection (Fig 6D). In addition, CD137 deletion on Tregs in *Tnfrsf9*^F/F^*Foxp3*^cre/+^ mice also led to a more severe CFU burden, increased levels of inflammatory factors, and more severe epithelial exfoliation compared to *Tnfrsf9*^F/F^*Foxp3*^+/+^ mice (Fig 6E-G). Given the established role of CD137L-CD137 reverse signaling, we compared the proportion of CD137L^+^ macrophages in the bladder following infection between *Tnfrsf9*^F/F^ *Foxp3*^cre/+^ and *Tnfrsf9*^F/F^ *Foxp3*^+/+^ mice. Surprisingly, no significant difference was detected between the two groups (S3 Fig).

## Discussion

Recent studies have provided some insights into the functions of immune cells, urothelial cells and antimicrobial peptides in the innate immune control of UPEC infection [32,40,41]. However, tissue-specific regulation of bladder immune responses to UPEC remains poorly understood. Urinary defense against invading pathogens relies on a complex immune cells network. While critical for pathogen clearance, the acute immune response can damage the urinary mucosa, increasing susceptibility to UTIs recurrence [42]. Moreover, the mechanisms driving inflammation resolution during UTIs are still poorly understood. The resolution of inflammation is an active process coordinated by mediators and immune cells to restore tissue homeostasis, crucial for preventing urinary pathogens and solutes from invading tissues and the bloodstream. The balance of activation and resolution of inflammation is under precise control. Improved understanding the mechanism of bladder immunomodulation will provide the infrastructure needed to pave the way for new immunotherapies to treat and prevent UTIs. To intensively investigate bladder immune microenvironment regulation during UTIs, we established a murine UPEC infection model and employed scRNA-seq to gain a novel, global view of the infected bladder’s immune landscape. Our research highlights the role of a specific subset of inflammation-associated macrophages, identified as CD137L^+^ macrophages, in suppressing acute inflammatory reactions by regulating Treg cells. These findings enhance our understanding of the inflammation resolution mechanisms in UPEC-infected bladders and lay the groundwork for developing immunotherapies targeting UTIs as well as other infectious diseases.

Macrophages, heterogeneous mononuclear phagocytes, play vital roles in host defense and tissue homeostasis [43]. Under stress and inflammation, they exhibit considerable context-dependent plasticity, rapidly responding to challenges within their environment and enter common and conserved polarized activation states that are observed across different tissues and organs [44]. Previous study has identified two unique subsets of macrophages reside in the bladder, directing the immune response to challenge UPEC infection [7]. The complexity of macrophage populations and responses during UTIs is highlighted by both established and emerging data, providing numerous avenues for further research into their temporal, spatial, and phenotypic dimensions. Our scRNA-seq analysis revealed a novel inflammation-associated macrophage subtype (CD137L^+^ macrophages) post-UPEC infection. Intriguingly, this subtype exert anti-inflammatory effects during UPEC infection.

CD137L is primarily expressed on professional APCs (dendritic cells, monocytes/macrophages, B cells), and its expression increases upon activation [45]. CD137L signaling has been characterized in the process of inflammation, hematopoiesis, and immune tolerance, with critical roles in multiple steps of inflammation progression [46] Here, we observed an increase in CD137L^+^ macrophages after UPEC infection. Several articles reported that macrophage depletion increased bacterial burden during primary infection [47,48], but it reduced bacterial burden during challenge infection [22]. Notably, LPS stimulation rapidly induces surface CD137L on macrophages, promoting pro-inflammatory polarization and sepsis through sustained inflammatory response [49,50]. In the present study, macrophage-specific CD137L deletion increased bacterial burden (CFU) and exacerbated bladder inflammation 24h post-infection. Although CD137L is required for sustained TNF production in macrophages [51]. TNF-α plays a crucial role in orchestrating neutrophil infiltration during bladder defense. And IL-1β is also a potent pro-inflammatory cytokine amplifying innate immune responses in several infectious diseases including urinary tract infections [52–54]. However, we detected no significant difference in IL-1β and TNF-α levels between *Tnfsf9*^F/F^*Lyz2*^Cre/+^ and *Tnfsf9*^F/F^*Lyz2*^+/+^ mice, which indicate that the exacerbation of inflammation upon macrophage CD137L deletion is not due to altered IL-1β/TNF-α production in macrophages.

To explore downstream mechanisms of CD137L^+^ macrophages, we analyzed immune cells, particularly lymphocytes, and observed a significant proliferation of Treg cells in the bladder of mice infected with UPEC accompanied by an up-regulated expression of CD137, which is the receptor for CD137L. Previous studies have reported infiltration of Treg into the bladder after primary UTIs [55], but their biological function remains undefined. Relevant to this, decreased immunosuppressive Treg are found in interstitial cystitis/bladder pain syndrome (IC/BPS) patients [56]. At least 50% of IC/BPS patients have a prior history of recurrent UTIs [57,58]. In view of these observations, Tregs may play an import role in the process of transition from recurrent UTIs to IC/BPS. Using a *Foxp3*DTR murine model of UPEC infection, we demonstrated that increased Tregs suppress acute bladder inflammation and protect urothelial integrity. Up-regulated pathways in CD137L^+^ macrophages, combined with Cellchat analysis, revealed that CD137L^+^ macrophages exhibit two reprogrammed functional states: (i) becoming inflammation-associated macrophages in UPEC-infected bladders, and (ii) specifically modulate Treg function at a subcellular level though CD137L-CD137 interaction.

The interaction between T lymphocytes and APCs, mediated by cytokines and direct cell contact, is crucial for initiating, regulating, and sustaining immunity against infection, as well as for controlling dysregulated immune responses. APCs present antigenic information to T lymphocytes. For antigen-dependent T cell activation to occur, APCs must provide not only the major histocompatibility complex (MHC)/T-cell receptor (TCR) signal but also engage costimulatory receptors with their ligands. CD137L serves as a context-dependent regulator of T-cell activation or inhibition [59], and its expression on APCs induces or upregulates CD137 expression on T cells [60]. CD137, a member of the tumor necrosis factor receptor superfamily, is primarily expressed on activated T cells [38]. CD137 signaling, in the presence of IL-2, promotes cell proliferation and survival of natural Tregs, enhancing their regulatory function [61]. Most studies indicate CD137^+^ Tregs possess stronger suppressive activity than CD137^−^ Tregs [62,63], while CD137 signaling may also convert Tregs to effector T cells (Teff) [62]. We found that Treg-specific CD137 deletion aggravated bacterial burden and bladder inflammation during UPEC infection. While *Tnfrsf9*^−/−^ mice exhibit no apparent abnormalities in the development of T lymphocytes and lymphoid organs; however, it is possible that interactions between CD137L and CD137 play a role in cellular development and differentiation [64]. The absence of CD137L^+^ macrophages exacerbated the intensity of UTIs, associated with a diminished population of Tregs and reduced CD137 expression on Tregs. Therefore, CD137^+^ Treg development appears dependent on CD137L^+^ macrophages, strongly suggesting that CD137L^+^ macrophages facilitate CD137^+^ Treg activity during acute infection.

The CD137L-CD137 interaction can activate ligand-dependent signal transduction pathways, known as “reverse signaling,” which elicits specific cellular responses [65]. Studies demonstrate that CD137 knockout in mice leads to hyperimmune responses and hyperproliferation of myeloid progenitors *in vitro* [65,66], while CD137L expression is upregulated on APCs [67]). Conversely, reverse signaling through CD137L, triggered by treating myeloid cells with recombinant CD137-Fc protein (rCD137-Fc), enhances myelopoiesis during inflammation [68,69]. *In vitro* studies further indicate that binding of CD137 on Tregs to CD137L on APCs leads to internalization of the CD137L-CD137 complex, thereby depriving APCs of immunostimulatory CD137L [70]. However, in our study using *Tnfrsf9*^F/F^*Foxp3*^cre/+^ mice with CD137 specifically deleted on Tregs, we observed no change in the proportion of CD137L^+^ macrophages. Considering that previous reports describe CD137L upregulation only upon systemic CD137 ablation or in isolated contexts, this differential effect strongly suggests a potential compensatory mechanism mediated by other CD137-expressing cell populations. Supporting this hypothesis, our transcriptomic analysis revealed CD137 expression in a subset of ILC2s, consistent with previous reports documenting CD137 on both intestinal and pulmonary ILC2s [71,72] and as confirmed by our prior work demonstrating bladder ILC2-mediated macrophage regulation [21]. This discrepancy also likely reflects the complex and context-dependent nature of immune responses mediated by the CD137L-CD137 signaling axis. Furthermore, CD137L expression levels on APCs may also be modulated by the strength and type of stimulus. Whether CD137^+^ Tregs can regulate CD137L^+^ macrophages during acute infection remains an open question. Additional experiments are required to delineate the contributions of these potential mechanisms.

In summary, this study revealed a protective CD137L^+^ macrophage population in the UPEC-infected bladder. Deletion of CD137L in macrophages resulted in severe bacterial burden and bladder inflammation during the acute stage of UPEC infection. The crucial role of CD137L^+^ macrophages in protecting against UPEC infection might be mediated by Tregs, which express high levels of CD137. Our results suggest that the CD137L-CD137 axis acts as a guardian of mucosal immunity during UTIs, protecting against immunopathology through macrophage-Treg coordination. Elucidating the molecular and cellular mechanisms of this protective acute checkpoint may transform clinical applications for UTI management and therapeutic strategies.

## Supporting information

S1 Fig Flow cytometry strategy. (A)Flow cytometry analysis strategy of CD45^+^ immune cells in bladder. (B) Flow cytometry analysis strategy of macrophages in bladder. (C) Flow cytometry analysis strategy of DCs in bladder. (D) Flow cytometry analysis strategy of Tregs in bladder.
(TIF)

S2 Fig Quality control of single cell sequencing data. Vlnplots show number of UMI (nUMI), number of genes (nGene) detected, percent of mitochondrial derived transcripts (percent.mito) and percent of ribosomal derived transcripts (percent.ribo) per single cell before and after quality control. (A) Control_1. (B) Control_2. (C) UPEC_1. (D) UPEC_2.
(TIF)

S3 Fig Treg-specific CD137 deletion does not alter the proportion of CD137L^+^ macrophages during UPEC bladder infection. CD137L^+^Ms in bladders from 8-week-old infected *Tnfrsf9*^*F/F*^ *Foxp3*^*Cre/+*^ and *Tnfrsf9*^*F/F*^ *Foxp3*^*+/+*^ mice were analyzed by flow cytometry. Dot plots depict the gating strategy for CD137L ^+^Ms. Graph shows the proportion of bladder CD137L^+^Ms (n = 4).
(TIF)

S1 Data The raw data supporting each of the manuscript Figures are contained in this Excel file. (XLSX)
