# Supplementary figures and images for: Single-cell analysis reveals an important role of CD137L+ macrophages in the host response to uropathogenic Escherichia coli infection in the bladder
Source: PLoS Pathog. 2025 Oct 3;21(10):e1013543. doi: 10.1371/journal.ppat.1013543 (PMC12494246; doi:10.1371/journal.ppat.1013543)

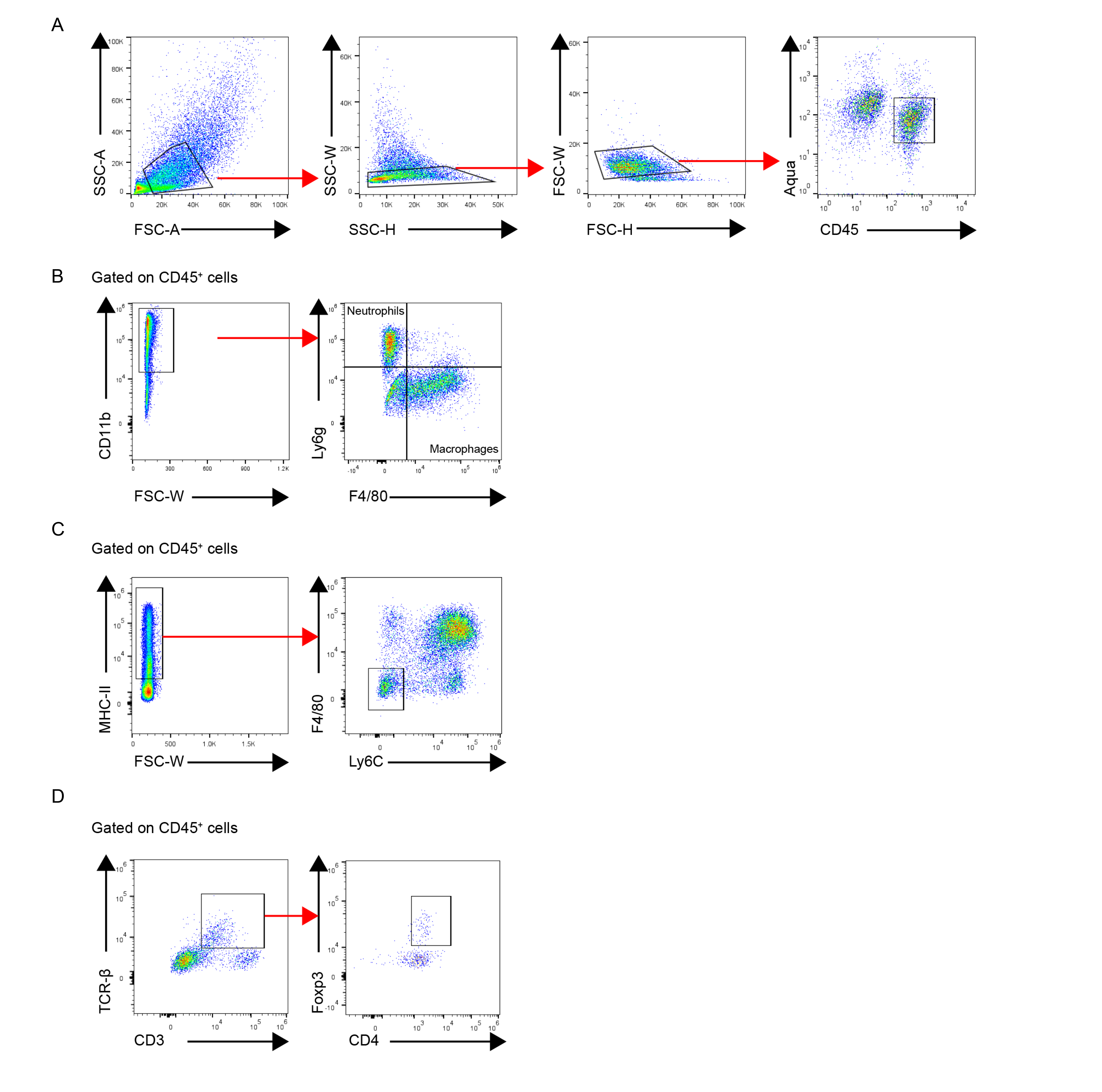

Supplement: S1 Fig — (A)Flow cytometry analysis strategy of CD45+ immune cells in bladder. (B) Flow cytometry analysis strategy of macrophages in bladder. (C) Flow cytometry analysis strategy of DCs in bladder. (D) Flow cytometry analysis strategy of Tregs in bladder. (TIF) [file ppat.1013543.s001.tif]

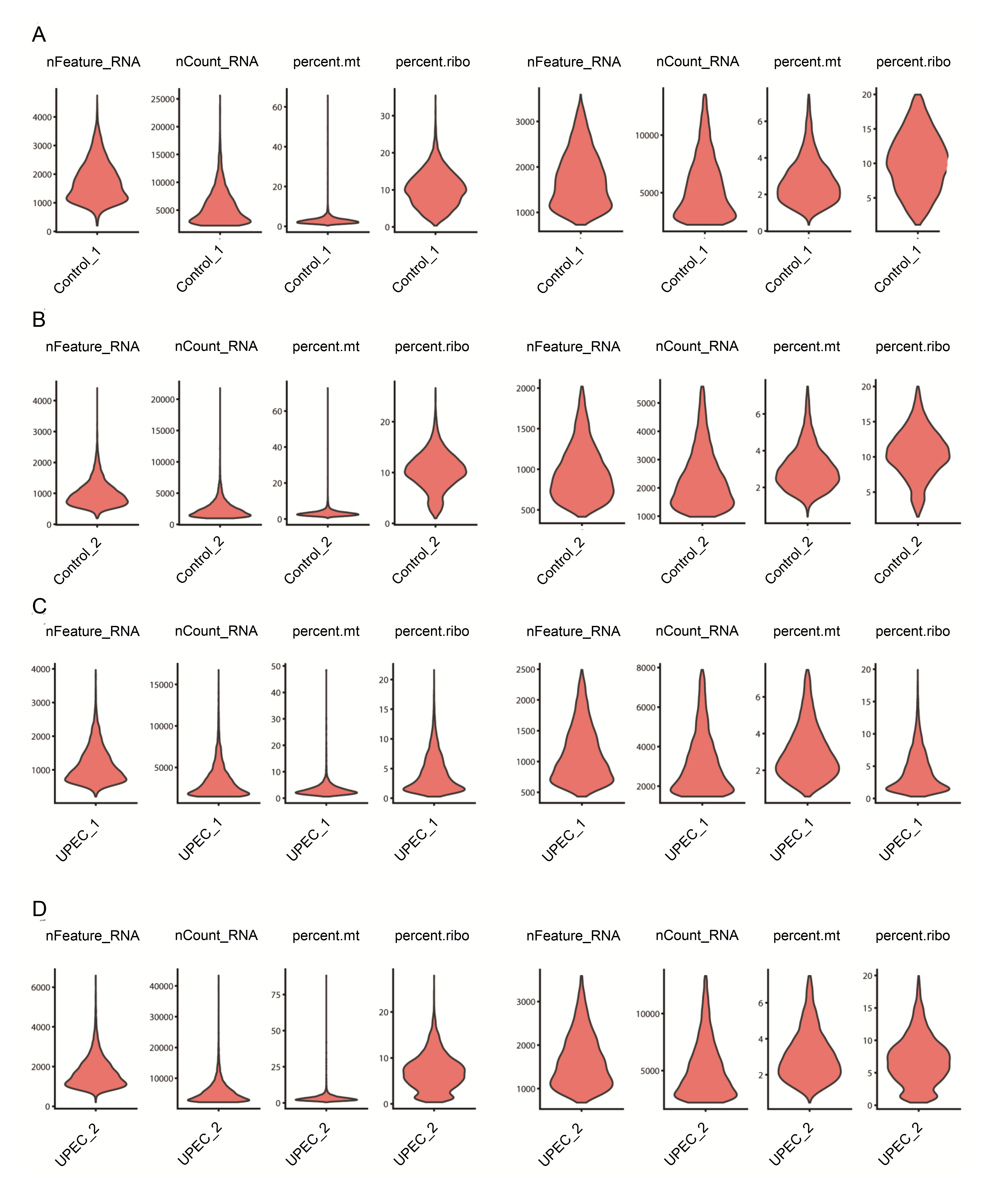

Supplement: S2 Fig — Vlnplots show number of UMI (nUMI), number of genes (nGene) detected, percent of mitochondrial derived transcripts (percent.mito) and percent of ribosomal derived transcripts (percent.ribo) per single cell before and after quality control. (A) Control_1. (B) Control_2. (C) UPEC_1. (D) UPEC_2. (TIF) [file ppat.1013543.s002.tif]

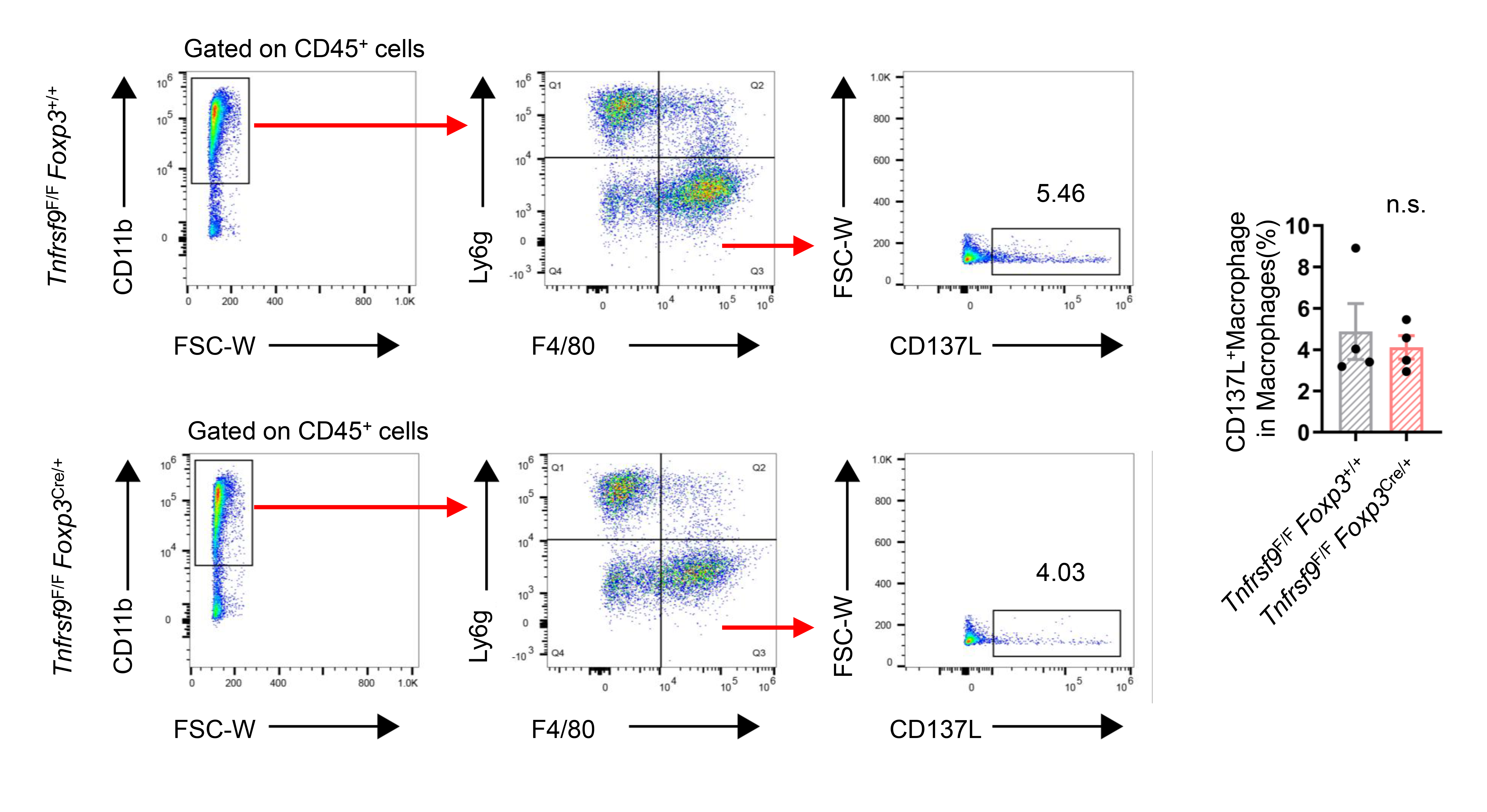

Supplement: S3 Fig — CD137L+Ms in bladders from 8-week-old infected Tnfrsf9F/F Foxp3Cre/+ and Tnfrsf9F/F Foxp3+/+ mice were analyzed by flow cytometry. Dot plots depict the gating strategy for CD137L +Ms. Graph shows the proportion of bladder CD137L+Ms (n = 4). (TIF) [file ppat.1013543.s003.tif]
